# Imaging xCT with Immuno-PET in Therapy-Resistant Non–Small Cell Lung Cancer

**DOI:** 10.2967/jnumed.126.272920

**Published:** 2026-10

**Authors:** Abigail R. Barber, Richard S. Edwards, Sofia N. dos Santos, Andrea Emanuelli, Will E. Tyrrell, Amaia Carrascal-Miniño, Kavitha Sunassee, Israt S. Alam, Gary J.R. Cook, Timothy H. Witney

**Affiliations:** 1 School of Biomedical Engineering and Imaging Sciences, King’s College London, St. Thomas’ Hospital, London, United Kingdom;; 2 Department of Radiology, Molecular Imaging Program at Stanford, Stanford University, Stanford, California; and; 3 Guy’s and St. Thomas’ PET Centre, St. Thomas’ Hospital, London, United Kingdom

**Keywords:** xCT, immuno-PET, therapy resistance, non–small cell lung cancer, molecular imaging

## Abstract

Therapy resistance presents a major clinical challenge in non–small cell lung cancer (NSCLC), contributing to its high mortality rate. Overexpression of the amino acid transporter xCT frequently mediates treatment resistance in NSCLC and can be therapeutically targeted with antibody–drug conjugates. Here, we developed a corresponding immuno-PET radiotracer to noninvasively evaluate xCT expression in NSCLC and visualize the pharmacokinetics of xCT-targeting antibodies. **Methods:** The specificity of xCT-targeting antibodies, HM30 and HM34, was assessed by Western blot, immunocytochemistry, and internalization studies. Next, these antibodies were conjugated to deferoxamine via their lysine residues and radiolabeled with ^89^Zr. Immunoreactivity was determined by bead- and cell-based binding assays. Preclinical in vivo pharmacokinetics of the ^89^Zr-labeled antibodies were evaluated through longitudinal imaging studies and ex vivo biodistribution using subcutaneous and orthotopic models of NSCLC. **Results:** Through multiple biochemical methods, we demonstrated excellent target specificity and rapid antibody internalization. Immuno-PET agents [^89^Zr]Zr-DFO-HM30 and [^89^Zr]Zr-DFO-HM34 were produced with good yield and radiochemical purity, with no impact on immunoreactivity. Both agents clearly delineated high xCT–expressing H460 tumors in vivo. Conversely, [^89^Zr]Zr-DFO-HM30 binding was low in low xCT–expressing H1299 tumors. [^89^Zr]Zr-DFO-HM30 imaging and autoradiography of H460 orthotopic lung tumors revealed lesion-specific binding. **Conclusion:** Our findings provide robust evidence for the use of immuno-PET as a research tool to image elevated xCT expression and visualize tumor penetration of xCT-directed antibodies. This tool can inform the development of enhanced antibody-based therapeutic candidates against treatment-resistant phenotypes to ultimately improve survival outcomes in patients with NSCLC.

## Visual Abstract

**Figure:**
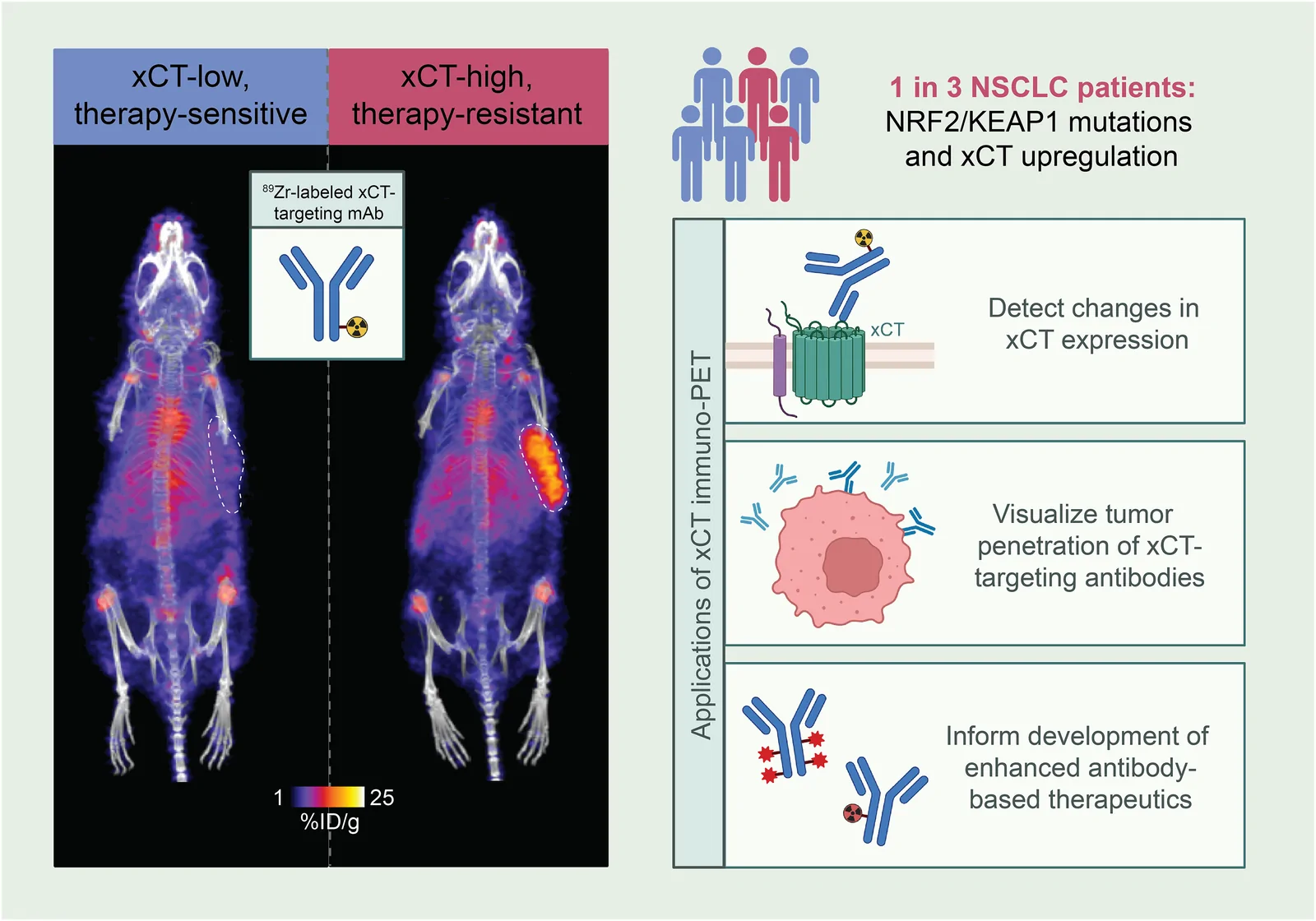

Outcomes for patients with non–small cell lung cancer (NSCLC) remain poor, with a 10-y survival rate of 11% (1). Patients are frequently diagnosed with late-stage disease, and resistance to standard-of-care therapy commonly arises. Antioxidant systems are essential for cancer cell adaptability, influencing tumor initiation, progression, and treatment resistance (2,3). Importantly, elevated antioxidant capacity enables tumors to withstand heightened oxidative stress arising from intrinsic metabolic activity and external therapeutic insult (4,5). Glutathione, a major intracellular antioxidant, is vital to maintain redox balance in NSCLC (6). Glutathione biosynthesis is increased in NSCLC by activating mutations in the transcription factor nuclear factor erythroid 2–related factor 2 (NRF2) or its negative regulator, kelch-like ECH-associated protein 1 (KEAP1), which occurs in approximately one third of patients with NSCLC (7). NRF2 activation directly drives glutathione synthesis through both the upregulation of biosynthetic enzymes and increased uptake of amino acids from the tumor microenvironment (8). One of these amino acids, cysteine, is rate-limiting for glutathione production (9) and is supplied largely via the cystine/glutamate antiporter, xCT (also known as SLC7A11). As xCT is under the transcriptional control of NRF2, targeting xCT offers a promising avenue to image the antioxidant capacity of tumors and treat resistant disease.

Despite the compelling role of xCT in cancer cell survival and therapy resistance, it has been underutilized as a therapeutic target. Past efforts have focused on the Food and Drug Administration–approved small-molecule xCT inhibitor sulfasalazine and its synergistic effect with platinum-based chemotherapies (10–12). Unfortunately, combination trials in humans have proved unsuccessful, largely because of low systemic bioavailability and insufficient specificity (13,14). To overcome these limitations, we previously investigated the targeted delivery of a cytotoxic payload to tumor cells using HM30-tesirine, an xCT-targeting antibody–drug conjugate (ADC). HM30-tesirine demonstrated robust inhibition of tumor growth and improved survival in a preclinical model of therapy-resistant NSCLC (15), highlighting the promise of antibody-based approaches targeting elevated xCT expression.

To fully realize the therapeutic potential of xCT-targeted biologics, a more rigorous understanding of antibody–target engagement and systemic pharmacokinetics is required. PET imaging offers a powerful, noninvasive approach to quantify target expression and engagement with high sensitivity. Several small-molecule PET radiotracers, such as (4*S*)-4-(3-[^18^F]fluoropropyl)-L-glutamate, (4*R*)-4-(3-[^18^F]fluoropropyl)-L-glutamate, [^18^F]hGST13, and [^18^F]fluoroaminosuberic acid, have been developed to visualize xCT function in vivo (16–19). Although providing valuable insights into treatment response and target expression (20–22), these small-molecule tracers cannot capture the target-binding properties and pharmacokinetics of antibody-based therapeutics against xCT. To address this limitation, we developed and tested [^89^Zr]Zr-DFO-HM30 and [^89^Zr]Zr-DFO-HM34 as immuno-PET companion diagnostics.

## MATERALS AND METHODS

Additional methods are provided in the supplemental materials, available at http://jnm.snmjournals.org.

### Conjugation and Radiolabeling

Lysine residues on HM30, HM34, and control human IgG1 (BioXcell) were labeled using *p*-SCN-Bn-deferoxamine (DFO; B-705; Macrocyclics). A previously published 2-step method, consisting of chelator–antibody conjugation and subsequent radiolabeling, was used (Supplemental Fig. 1) (23). Briefly, a 5-fold molar excess of *p*-SCN-Bn-DFO in 20 µL of dimethyl sulfoxide (4–7 mM, 3–5 mg/mL) was added to 2–4 mg of antibody (1 mL) and incubated for 1 h (37 °C, 550 rpm). The reaction product was then purified using a PD-10 column (Cytiva) and eluted with phosphate-buffered saline (PBS) (2 mL).

For radiolabeling, ^89^Zr in 1M oxalic acid (Revvity) was neutralized with 2M sodium carbonate before incubation with DFO-conjugated antibodies (DFO-Abs) in 0.5M 4-(2-hydroxyethyl)-1-piperazineethanesulfonic acid buffer for 1 h at room temperature (550 rpm). For in vitro productions, 4–8 MBq of ^89^Zr was added to 20 µg of DFO-Abs. For in vivo productions, 40–80 MBq of ^89^Zr was added to 200–500 µg of DFO-Abs. [^89^Zr]Zr-DFO-Abs were purified using a PD-10 column and eluted with PBS (2 mL) in four 500-µL fractions. Radiochemical conversion and radiochemical purity were assessed as detailed in the supplemental information. Radiolabeling yields have not been decay-corrected.

### Bead-Based Immunoreactivity

The immunoreactivity of [^89^Zr]Zr-DFO-HM30/34 was measured by a bead-based assay, as described by Sharma et al. (24). Briefly, 20 µL of DynaBeads MyOne Streptavidin-T1 (Invitrogen) were washed by adding 380 µL of PBS with 0.05% Tween20, followed by brief vortexing. Beads were isolated using the DynaMag magnetic rack (Invitrogen) before incubation with 2 µg of biotinylated xCT antigen (amino acid sequence, KGQTQNFKDAFSGRDSSITRLP) for 15 min with end-to-end rotation. After antigen loading, the beads were washed 3 times. Next, 1 ng of [^89^Zr]Zr-DFO-HM30/34 (∼250 Bq), with and without 5 µg of unlabeled antibody, were added to the beads and incubated for 30 min at room temperature with rotation. The beads were isolated, and the supernatant was collected in fresh Eppendorf tubes. The beads were then washed twice with PBS with 0.05% Tween20 while also collecting the washes in fresh Eppendorf tubes. Finally, radioactivity in the supernatant, washes, and beads were measured in a γ-counter (LKB Wallac 1282 CompuGamma; PerkinElmer). Immunoreactivity was calculated using the following equation:

$$\text{Immunoreactivity}\;(\%\;\text{total}\;\text{activity})=\left(\frac{\text{bead}\;\text{CPM}}{\text{supernatant}\;\text{CPM}+\text{washes}\;\text{CPM}+\text{bead}\;\text{CPM}}\right)\times 100,$$ Eq. 1

where CPM denotes counts per minute.

### Animal Studies

All animal experiments were performed in accordance with the United Kingdom Home Office Animal (scientific procedures) Act 1986 under project license PP9982297 and received local Animal Welfare and Ethical Review Body approval.

### Longitudinal PET/CT Imaging

Female BALB/C *nu/nu* mice were anesthetized with 1%–2% isoflurane in O_2_ before intravenous injection through a tail vein cannula of 10 µg of [^89^Zr]Zr-DFO-Abs (∼2 MBq), with and without 90 µg of unlabeled DFO-Abs, in 100 µL of PBS. Static PET images were acquired at 4, 24, 48, 72, 96, and 144 h postinjection on a Mediso NanoScan PET/CT system (1–5 coincidence mode, 3-dimensional reconstruction with CT-based attenuation and scatter corrections) using a 4-bed mouse hotel (25). Scan time was increased at each time point to account for the half-life of ^89^Zr (3.3 d), ranging from 30 to 90 min. CT images were acquired for anatomic visualization and attenuation correction (720 projections, helical acquisition, 50 kV, 300 ms exposure). Static PET image reconstruction was performed using the 3-dimensional Tera-Tomo algorithm (4 iterations, 6 subsets, 0.4 mm isotropic voxel size, 400- to 600-keV energy window). InterviewFusion software version 3.11.011 (Mediso) was used for automatic 4-bed hotel separation. DICOM data for individual mice were imported into VivoQuant software version 5.4.2 (Perceptive) for image quantification. Tissue volumes of interest were compiled from manually drawn sequential 2-dimensional regions of interest from the CT images. The radioactivity in each tissue was expressed as a percentage of the injected dose per gram of tissue volume.

### Ex Vivo Biodistribution

After completion of the final PET/CT scans, mice were sacrificed, and tissues of interest were harvested and weighed. The radioactivity in each tissue was measured using a γ-counter (LKB Wallac 1282 CompuGamma). To allow for the accurate quantification of the radioactivity, serially diluted ^89^Zr standards were measured in parallel. Radioactivity measurements were decay-corrected to the time of injection and normalized to tissue weight.

### Statistical Analysis

Data are expressed as mean ± SD. Significance was determined by ordinary 1-way ANOVA with Dunnett or Tukey post hoc test, 2-way ANOVA with Bonferroni adjustment for multiple comparisons, or Welch *t* test (Prism version 10.3.1, GraphPad Software). A *P* value of less than 0.05 was considered significant.

## RESULTS

### Monoclonal Antibodies (mAbs) HM30 and HM34 Selectively Bind to xCT

The mAbs used in this study, HM30 and HM34, were provided by AgilVax (patent WO2020/227640A1). Both are humanized IgG1 antibodies that target human xCT at an epitope within its extracellular domain 3 (Fig. 1A). To confirm target specificity, a panel of human NSCLC cell lines with varying xCT expression were probed by Western blot using these antibodies. HM30 and HM34 differentiated between high and low xCT–expressing cells, with the magnitude of xCT expression matching data generated using a commercially available antibody (Fig. 1B; Supplemental Fig. 2). Surface plasmon resonance also confirmed the high affinity of antibodies for their target antigen (*K*_D_ = 0.08 nM and 0.11 nM for HM30 and HM34, respectively; Supplemental Fig. 3). Cell-membrane binding of HM30 and HM34 was confirmed by confocal microscopy in xCT-high H460 cells, with staining colocalized with membrane-specific CellBrite fluorescence (Fig. 1C). Further, HM30 and HM34 mAbs were rapidly internalized by H460 cells, with pHrodo Red (Invitrogen) fluorescence of labeled mAbs increasing in a time-dependent manner after activation in the acidic environment of endosomes and lysosomes (Fig. 1D).

**FIGURE 1.**
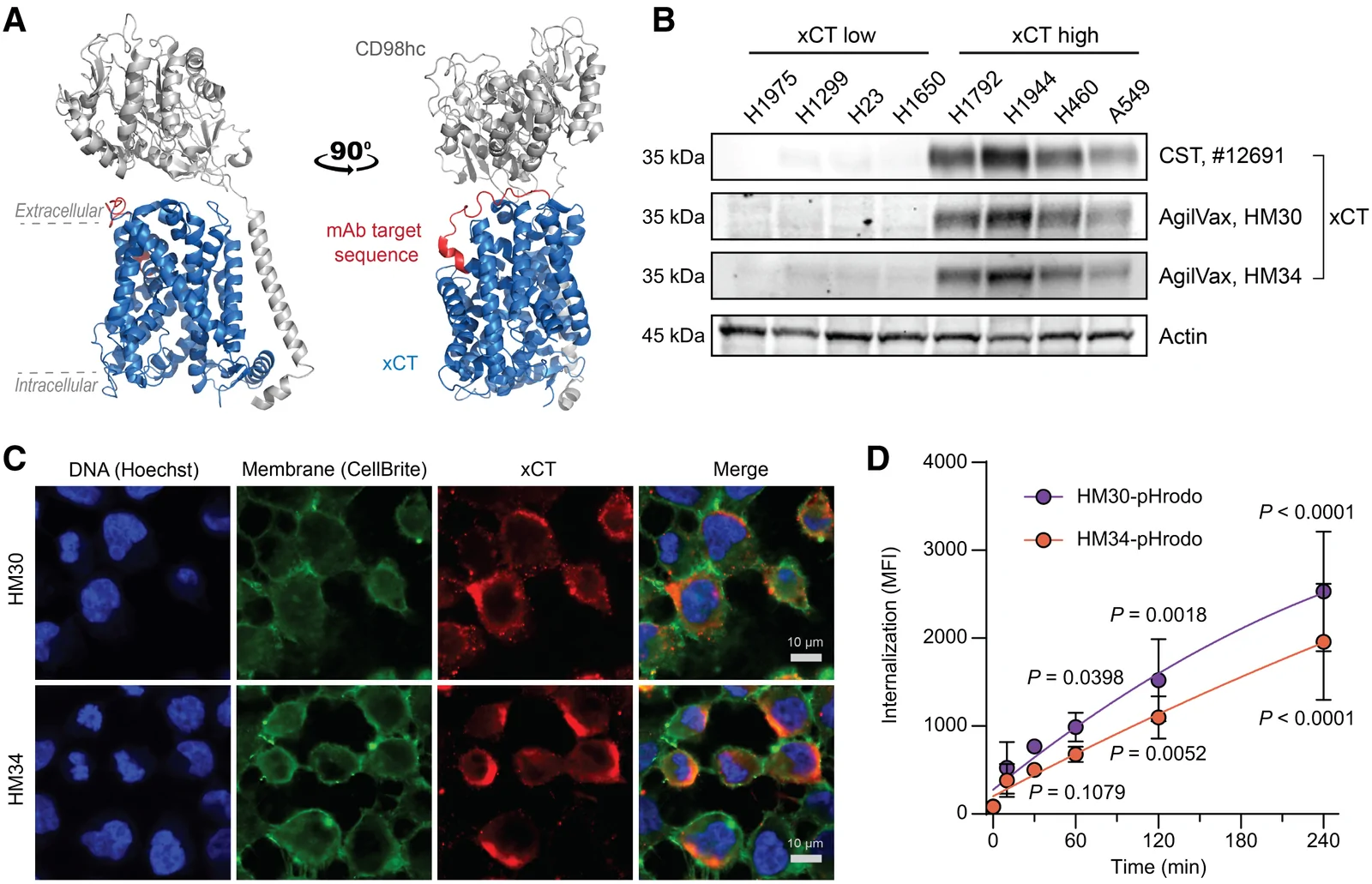
Specificity of HM30 and HM34 for xCT. (A) Protein structure of xCT (blue) and its associated CD98 heavy chain (CD98hc, gray), required for membrane localization (Protein Data Bank ID: 7EPZ) (30). Antibody target sequence is highlighted in red. (B) xCT expression determined by Western blot using HM30 and HM34 as primary antibodies, alongside commercial antibody, Cell Signaling Technology (CST) #12691. Actin was used as loading control. (C) Immunocytochemistry with H460 cells, visualizing membrane localization of HM30 and HM34 staining. (D) Flow cytometric assessment of pHrodo Red-labeled HM30 and HM34 internalization in H460 cells over 4 h (*n* = 3); comparisons with 0 min were made by 1-way ANOVA with Dunnett post hoc test. MFI = median fluorescence intensity.

### Radiolabeling of HM30 and HM34 Does Not Affect Immunoreactivity

Mass spectrometry revealed a chelate-to-antibody ratio of 1.84 for DFO-HM30 and 1.72 for DFO-HM34. Importantly, DFO conjugation to HM30 and HM34 had no impact on xCT binding, as shown by flow cytometry and immunoprecipitation assays (Fig. 2A; Supplemental Fig. 4). Subsequent radiolabeling produced [^89^Zr]Zr-DFO-HM30 and [^89^Zr]Zr-DFO-HM34 in yields of 82.5% ± 12.4% (*n* = 4) and 94.8% ± 5.4% (*n* = 5), respectively, with high radiochemical purity (98.1% ± 1.6% for [^89^Zr]Zr-DFO-HM30, *n* = 4; 97.3 ± 1.0% for [^89^Zr]Zr-DFO-HM34, *n* = 5). Radiochemical purity was confirmed by fast protein liquid chromatography and subsequent γ-counting, with antibody integrity confirmed via sodium dodecyl sulfate polyacrylamide gel electrophoresis with phosphoimaging (Supplemental Fig. 5). The specific activities of the radioimmunoconjugates were 0.225 ± 0.090 MBq/µg (*n* = 4) and 0.268 ± 0.089 MBq/µg (*n* = 5) for [^89^Zr]Zr-DFO-HM30 and [^89^Zr]Zr-DFO-HM34, respectively.

**FIGURE 2.**
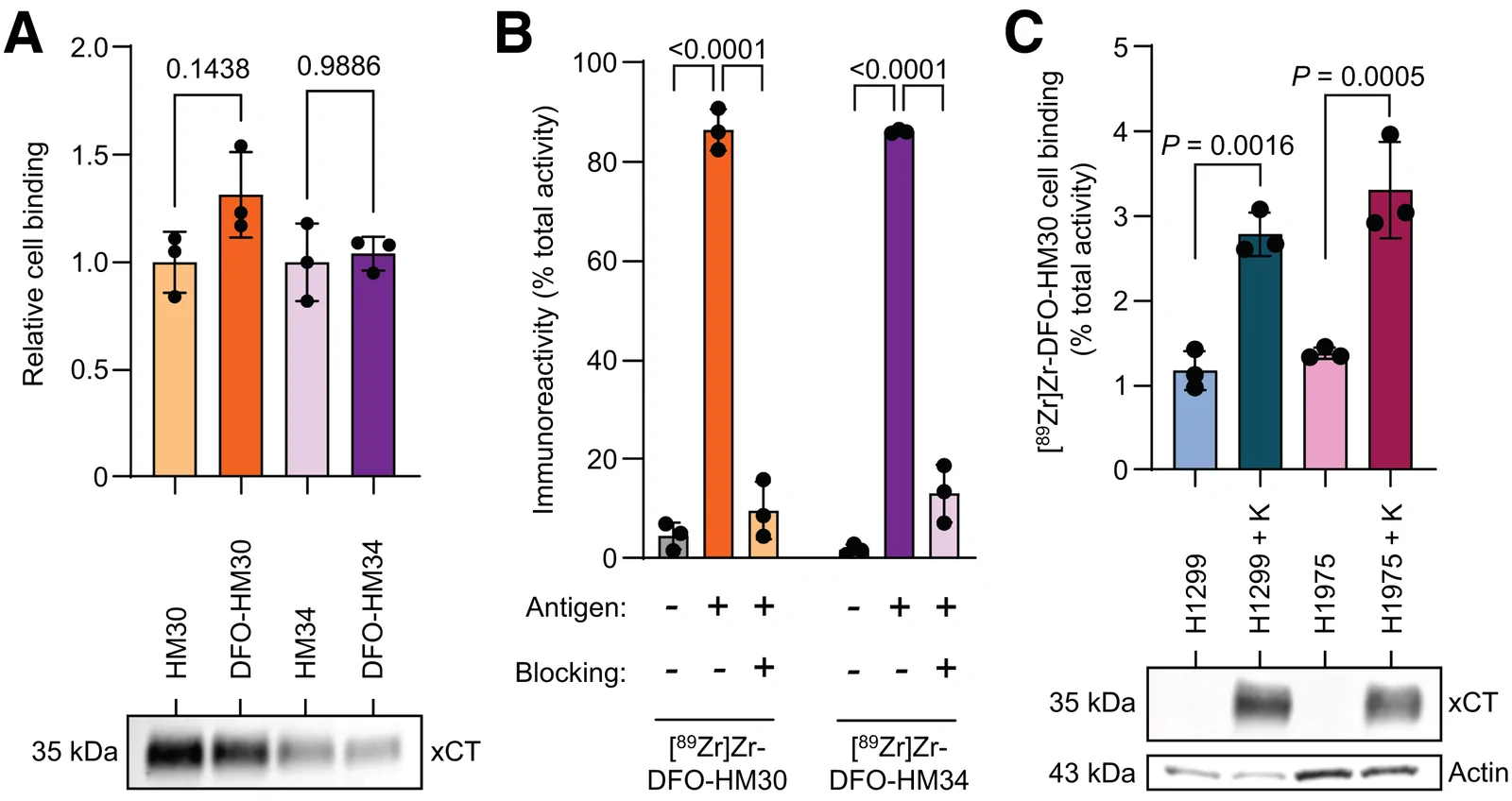
Immunoreactivity of [^89^Zr]Zr-DFO-HM30 and [^89^Zr]Zr-DFO-HM34. (A) (Top) Flow cytometric analysis of unconjugated vs. DFO-conjugated HM30 and HM34 binding to H460 cells (*n* = 3). Median fluorescence intensity (MFI) of each mAb was normalized to average MFI of their respective unconjugated mAbs to yield relative cell binding values. (Bottom) Western blot after immunoprecipitation of xCT from H460 lysate, comparing unconjugated and DFO-Abs. Detection was performed with commercial antibody. (B) Bead-based immunoreactivity assay of ^89^Zr-labeled antibodies with immobilized target peptide ± 5000-fold excess of corresponding unlabeled mAb (*n* = 3). Unloaded beads were included as control. (C) (Top) Cell-binding of [^89^Zr]Zr-DFO-HM30 in NSCLC cell lines before and after treatment with 200 µm of ki696. (Bottom) xCT expression after ki696 treatment. Actin was included as loading control. All comparisons were made using 1-way ANOVA with Tukey post hoc test.

After radiolabeling, a bead-based antigen-binding assay was performed to determine antibody immunoreactivity. Both [^89^Zr]Zr-DFO-HM30 and [^89^Zr]Zr-DFO-HM34 had high immunoreactivity to the target antigen (86.4% and 86.1%, respectively; *P* < 0.0001) compared with unloaded control beads (Fig. 2B). Notably, the binding of the radiolabeled mAbs to the target antigen was blocked by more than 70% when coincubated with a 5000-fold excess of the corresponding unlabeled mAb (*P* < 0.0001, *n* = 3; Fig. 2B), further indicating specificity for xCT. Finally, [^89^Zr]Zr-DFO-HM30 was evaluated in H1299 and H1975 NSCLC cells, which exhibit low basal xCT expression. Treatment of these cells with ki696, an NRF2 pathway activator (15), increased xCT expression and [^89^Zr]Zr-DFO-HM30 cell binding by 2.4-fold compared with untreated controls (*P* = 0.0016 and *P* = 0.0005 for H1299 and H1975 cells, respectively (Fig. 2C).

### [^89^Zr]Zr-DFO-HM30 and [^89^Zr]Zr-DFO-HM34 Bind to NSCLC Tumors In Vivo

To evaluate the ability of [^89^Zr]Zr-DFO-HM30 and [^89^Zr]Zr-DFO-HM34 to identify xCT-expressing NSCLC tumors, longitudinal PET/CT imaging was performed in female mice bearing subcutaneous H460 tumors. At 4 h postinjection, both [^89^Zr]Zr-DFO-HM30 and [^89^Zr]Zr-DFO-HM34 remained in the circulation, illustrated by high levels of radioactivity in the heart, carotid arteries, and descending aorta (Fig. 3A; Supplemental Fig. 6). Tumor accumulation of both agents was present at 24 h postinjection, and there were no differences in tumor binding between [^89^Zr]Zr-DFO-HM30 and [^89^Zr]Zr-DFO-HM34 at any of the time points tested (*P* > 0.05, *n* = 4 (Fig. 3B). Ex vivo biodistribution studies confirmed tumor binding of both agents at 96 h postinjection (10.34 %ID/g ± 0.60 %ID/g for [^89^Zr]Zr-DFO-HM30 and 7.54 %ID/g ± 1.23 %ID/g with [^89^Zr]Zr-DFO-HM34 (Fig. 3C). Off-target accumulation was observed in the liver, spleen, and knee and shoulder joints, the latter of which likely reflects a small degree of ^89^Zr^4+^ dissociation from DFO (Fig. 3A) (26). Ex vivo biodistribution studies confirmed that both [^89^Zr]Zr-DFO-HM30 and [^89^Zr]Zr-DFO-HM34 levels remained high in the blood pool at 96 h postinjection (Fig. 3C).

**FIGURE 3.**
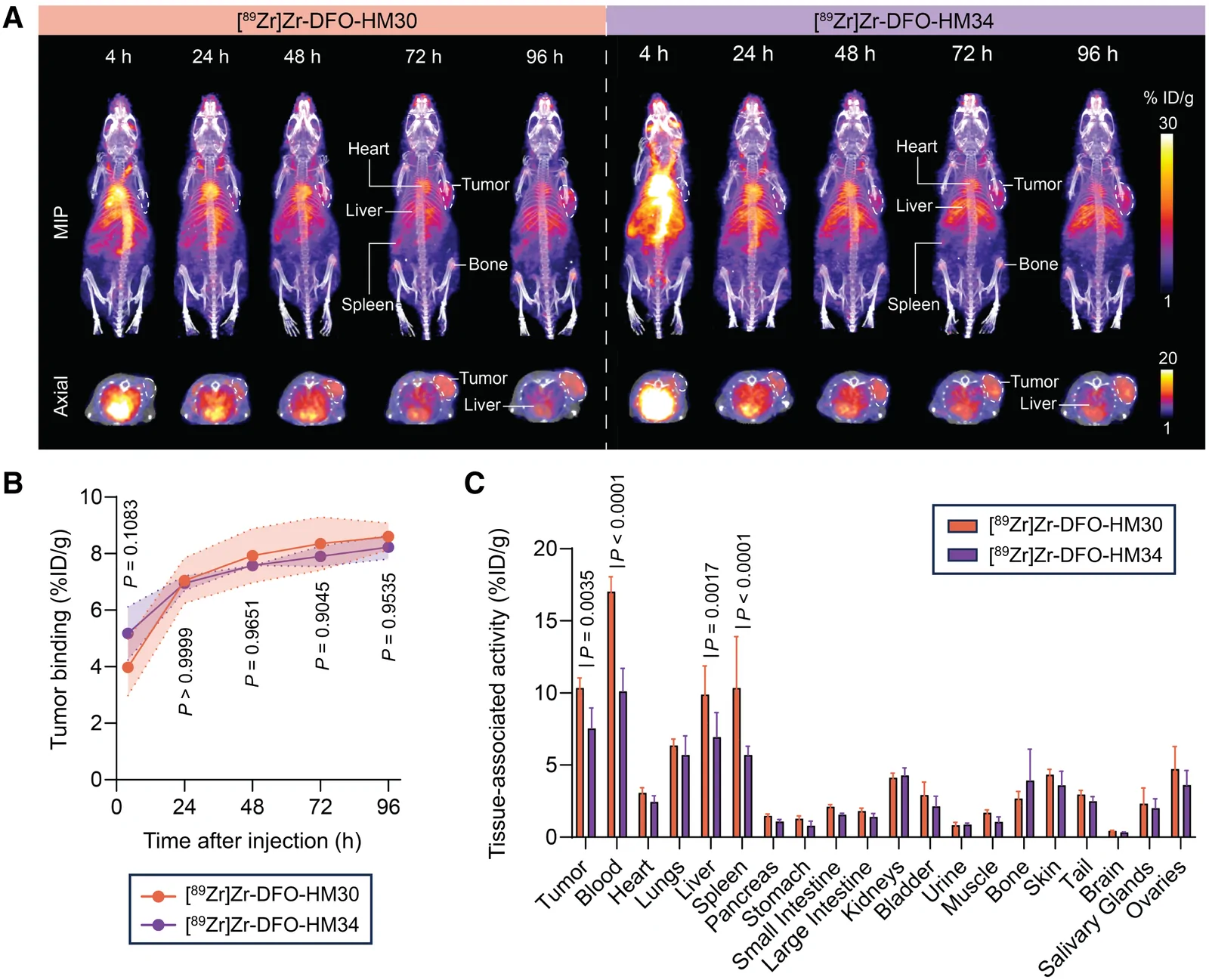
Evaluation of [^89^Zr]Zr-DFO-HM30 and [^89^Zr]Zr-DFO-HM34 in H460 tumor–bearing mice. (A) Maximum-intensity-projection (MIP, top) and axial slice (bottom) PET/CT images of BALB/c *nu/nu* mice bearing subcutaneous H460 tumors at 4, 24, 48, 72, and 96 h postinjection of 10 μg of [^89^Zr]Zr-DFO-HM30 or [^89^Zr]Zr-DFO-HM34. (B) Tumor image quantification (*n* = 4). (C) Ex vivo biodistribution with [^89^Zr]Zr-DFO-HM30 and [^89^Zr]Zr-DFO-HM34 at 96 h postinjection. Values are expressed as average % ID/g ± SD (*n* = 4). Comparisons were made using 2-way ANOVA with Bonferroni adjustment for multiple comparisons.

In parallel, a second cohort of mice was coinjected with an additional 90 µg of unlabeled HM30 or HM34 alongside 10 µg of corresponding [^89^Zr]Zr-DFO-HM30/34 to better understand whether accumulation in healthy tissues can be blocked without impacting tumor binding. In both cases, injection with or without coinjection of 90 µg of unlabeled mAb made no difference in accumulation in the liver (*P* > 0.99 and *P* = 0.53) or spleen (*P* > 0.99 and *P* = 0.21) 96 h after injection, with [^89^Zr]Zr-DFO-HM30 and [^89^Zr]Zr-DFO-HM34, respectively (Supplemental Figs. 7 and 8). The estimated effective doses for [^89^Zr]Zr-DFO-HM30 and [^89^Zr]Zr-DFO-HM34, calculated by mouse-to-human extrapolated dosimetry, were comparable, at 0.34 and 0.38 mSv/MBq, respectively. The highest absorbed doses were observed in the liver (1.48 and 1.67 mGy/MBq) and kidneys (1.08 and 1.04 mGy/MBq for [^89^Zr]Zr-DFO-HM30 and [^89^Zr]Zr-DFO-HM34, respectively (Supplemental Table 1).

### [^89^Zr]Zr-DFO-HM30 Immuno-PET Can Distinguish xCT Expression In Vivo

To confirm that tumor accumulation was xCT-specific, we next performed imaging with [^89^Zr]Zr-DFO-HM30 in female mice bearing either H1299 (xCT-low) or H460 (xCT-high) subcutaneous tumors. As both radioimmunoconjugates performed similarly in vivo (Fig. 3), [^89^Zr]Zr-DFO-HM30 was used to contextualize our previous study, which used HM30-tesirine in the same tumor models (15). At 144 h postinjection of [^89^Zr]Zr-DFO-HM30, H460 tumors were clearly visualized by PET/CT imaging, whereas H1299 tumors were not visible above background activity (Fig. 4A; Supplemental Fig. 9). The extent of nonspecific tumor binding was visualized using a control IgG1 antibody, [^89^Zr]Zr-DFO-hIgG1, in H460 tumor-bearing mice (Fig. 4A; Supplemental Fig. 9). Differences in tumor accumulation were quantified by ex vivo biodistribution at 144 h, where [^89^Zr]Zr-DFO-HM30 binding was 2.1-fold higher than [^89^Zr]Zr-DFO-hIgG1 (10.83 %ID/g ± 2.30 %ID/g and 5.04 %ID/g ± 0.61 %ID/g, respectively, *P* < 0.0001; Fig. 4B), confirming specificity for xCT. Similarly, H460 (xCT-high) tumors had 2.7-fold higher [^89^Zr]Zr-DFO-HM30 binding than H1299 (xCT-low) tumors (4.05 %ID/g ± 1.01 %ID/g, *P* < 0.0001) (Fig. 4B). Ex vivo autoradiograms confirmed higher [^89^Zr]Zr-DFO-HM30 accumulation in H460 tumors compared with H1299 tumors, corresponding with xCT expression levels (Supplemental Fig. 10). There were no differences in [^89^Zr]Zr-DFO-HM30 accumulation in off-target organs, irrespective of the tumor type (Fig. 4B). [^89^Zr]Zr-DFO-hIgG1 had reduced accumulation in the lungs compared with [^89^Zr]Zr-DFO-HM30 (*P* < 0.0001), likely because of its faster blood clearance (*P* < 0.0001) (Fig. 4). In contrast, the [^89^Zr]Zr-DFO-hIgG1 signal in the spleen and kidneys was elevated compared with that of [^89^Zr]Zr-DFO-HM30 (*P* = 0.0117 and *P* = 0.0007, respectively (Fig. 4B).

**FIGURE 4.**
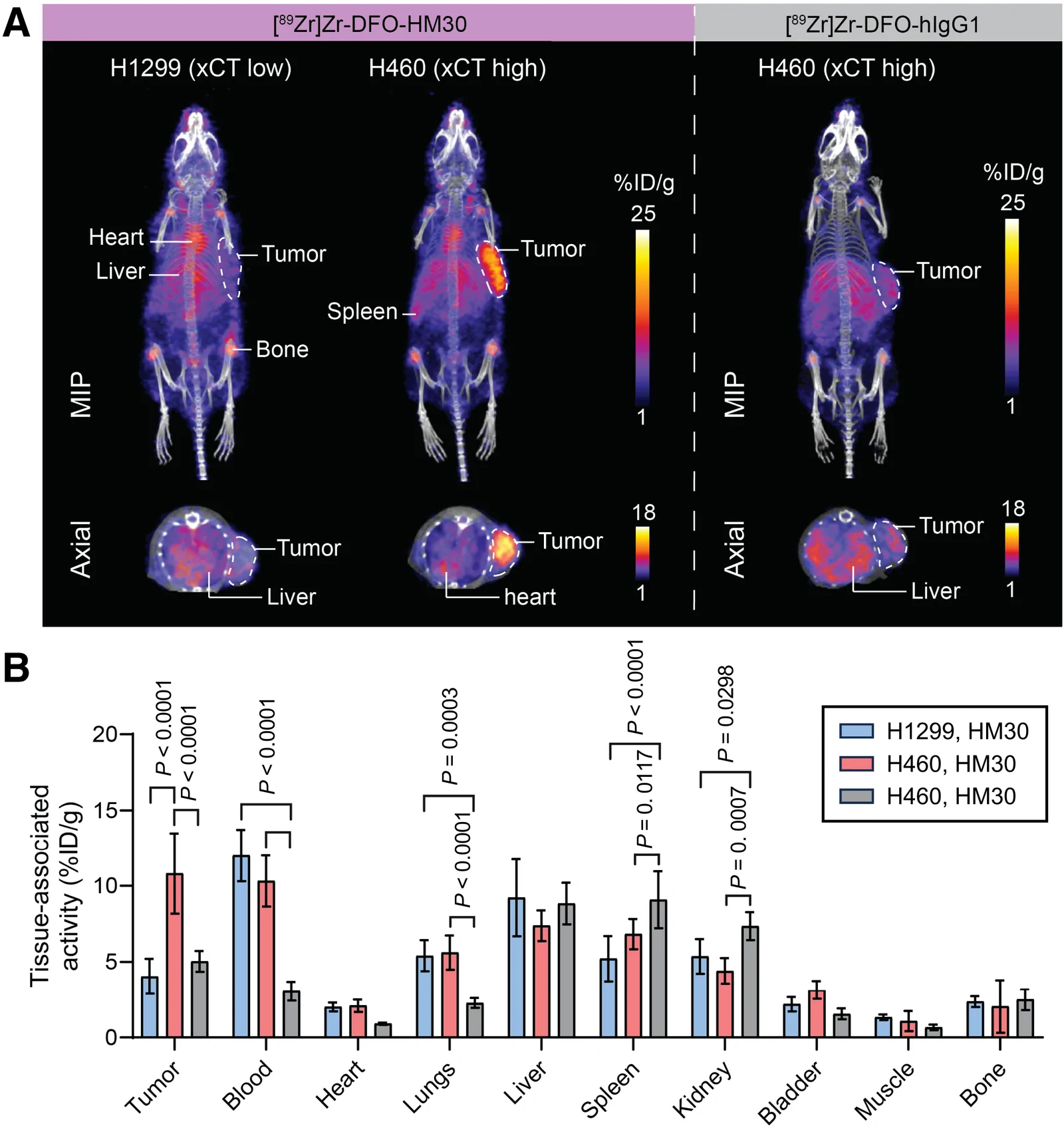
[^89^Zr]Zr-DFO-HM30 differentiates xCT-high from xCT-low tumors. (A) Representative maximum-intensity-projection (MIP; top) and axial slice (bottom) PET/CT images of BALB/c *nu/nu* mice bearing subcutaneous H1299 or H460 tumors at 144 h postinjection of 10 μg of [^89^Zr]Zr-DFO-HM30 or [^89^Zr]Zr-DFO-hIgG1. (B) Corresponding ex vivo biodistribution from selected tissues. Values are expressed as average % ID/g ± SD (*n* = 4). Comparisons were made using 2-way ANOVA with Bonferroni adjustment for multiple comparisons.

### [^89^Zr]Zr-DFO-HM30 Immuno-PET Can Visualize Orthotopic Lung Tumors In Vivo

To investigate antibody-based xCT imaging in a more clinically relevant model, xCT-high H460 tumors were grown in the lungs of mice, with [^89^Zr]Zr-DFO-HM30 imaging performed 6 d after [^89^Zr]Zr-DFO-HM30 administration. [^89^Zr]Zr-DFO-HM30 accumulation in H460 lung tumors was significantly higher than in healthy lungs (7.81 %ID/g ± 1.23 %ID/g vs. 4.65 %ID/g ± 0.34 %ID/g, respectively; *P* < 0.0001) (Fig. 5A; Supplemental Fig. 11). Image analysis revealed a degree of interlesion heterogeneity in [^89^Zr]Zr-DFO-HM30 binding, both within and between mice (Fig. 5A; Supplemental Fig. 11). Ex vivo autoradiography and immunohistochemical staining of tumor and lung tissue confirmed that [^89^Zr]Zr-DFO-HM30 accumulation was tumor-specific and corresponded with high xCT expression (Fig. 5B). Interestingly, animals with tumors had marginally higher accumulation of [^89^Zr]Zr-DFO-HM30 in the heart and spleen compared with healthy animals (*P* = 0.0331 and *P* = 0.0390, respectively) (Supplemental Fig. 12), suggesting prolonged blood circulation in this disease model.

**FIGURE 5.**
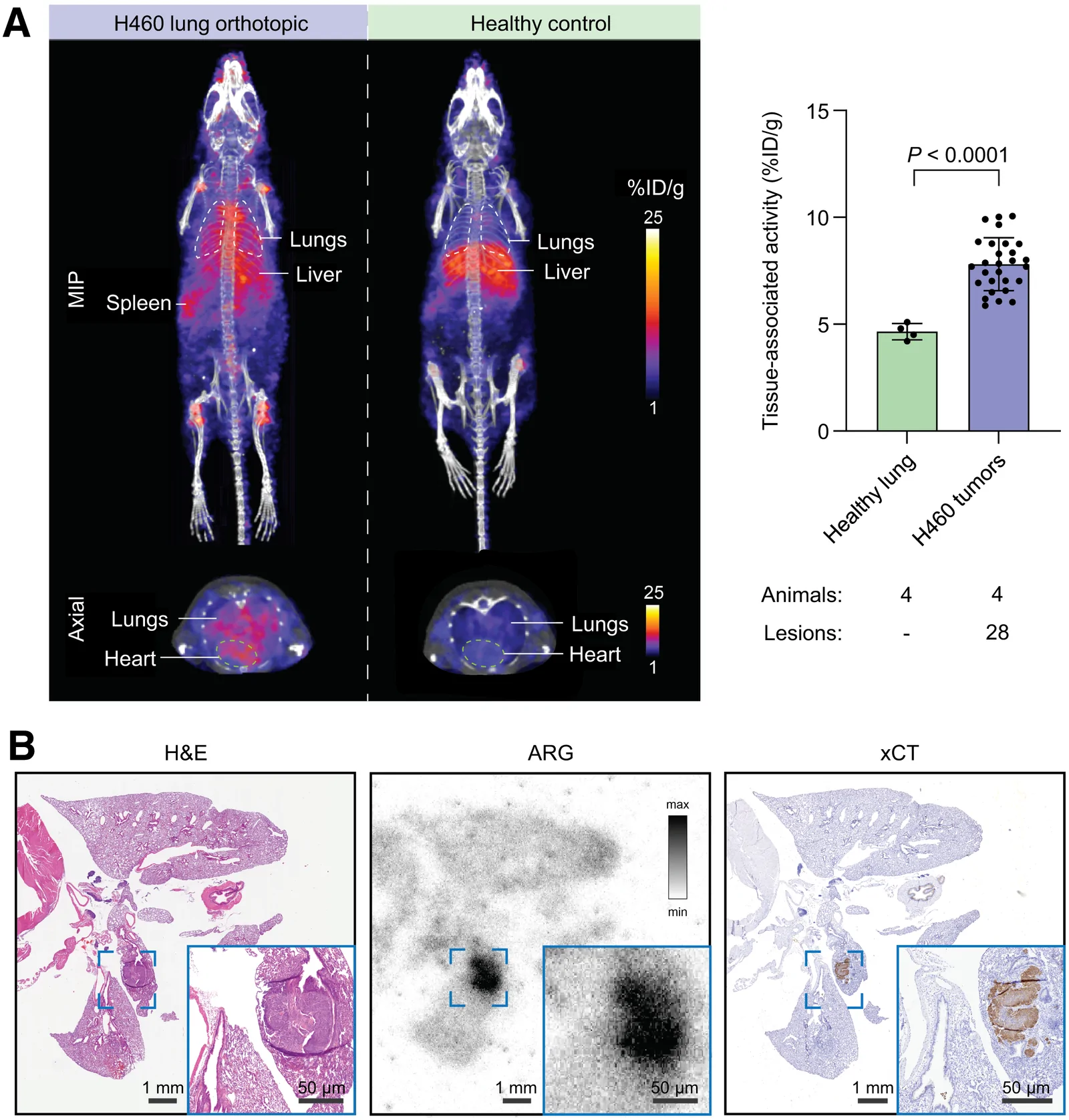
Visualization of xCT-high lung tumors with [^89^Zr]Zr-DFO-HM30. (A) Representative maximum-intensity-projection (MIP; top) and axial slice (bottom) PET/CT images of BALB/c *nu/nu* mice bearing lung orthotopic H460 tumors or healthy control animals at 144 h postinjection of 10 μg of [^89^Zr]Zr-DFO-HM30. Image quantification of [^89^Zr]Zr-DFO-HM30 tumor accumulation compared with lungs of healthy controls. Values are mean ± SD. Comparisons were made using Welch *t* test. (B) Representative postimaging autoradiogram (ARG), illustrating [^89^Zr]Zr-DFO-HM30 accumulation, alongside corresponding hematoxylin and eosin (H&E)- and xCT-stained tissue sections.

## DISCUSSION

Alterations in tumor redox metabolism bolster antioxidant defenses and promote resistance to traditional therapeutics. Central to this mechanism is the cell’s predominant antioxidant, glutathione, whose increased production is facilitated by upregulated expression of xCT via NRF2 activation. Here, we showed that a humanized antibody can noninvasively image xCT expression in vivo, visualizing a therapeutically targetable resistance mechanism with immuno-PET.

Antibody-based therapeutics targeting xCT have shown promise in NSCLC (15), but their in vivo behavior requires further characterization. In this study, [^89^Zr]Zr-DFO-HM30 and [^89^Zr]Zr-DFO-HM34 enabled immuno-PET visualization of the tumor-binding abilities and pharmacokinetics of xCT-directed antibodies. Both tracers exhibited characteristic immuno-PET in vivo biodistribution, including prolonged blood circulation and accumulation in the liver and spleen, which may reflect the preserved binding affinity of humanized IgG to murine fragment crystallizable γ-receptors in BALB/c *nu/nu* mice (27,28). Here, [^89^Zr]Zr-DFO-HM30 demonstrated xCT-specific tumor binding in both subcutaneous and orthotopic NSCLC models, providing an invaluable opportunity to study tumor penetration and off-target accumulation of xCT-targeting therapeutic antibodies.

Immuno-PET agents hold great value as diagnostic agents through their high-affinity antigen binding. The antibodies tested here bound xCT with subnanomolar *K*_D_ and were rapidly internalized. Tumor binding of [^89^Zr]Zr-DFO-HM30, however, did not exceed 11 %ID/g at 144 h postinjection, despite remaining high in circulation. Interestingly, the level of in vivo tumor binding observed with our immuno-PET agents matches that reported in a previous immuno-PET study targeting LAT1 in colorectal cancer (29). LAT1 and xCT are membrane-localized by CD98hc, whose bulky ectodomain partially obstructs xCT’s small extracellular binding site (30,31), potentially limiting antibody access, thereby reducing contrast. Tumor-to-background ratios could be improved by mapping accessible epitopes to guide the development of next-generation xCT-targeting antibodies. Additionally, targeting xCT with smaller biologics, such as single-domain antibodies or nanobodies, could circumvent potential antigen inaccessibility.

Although this study describes an xCT immuno-PET agent with high xCT affinity and a potential for use as a companion diagnostic, the affinity of mAbs for murine xCT is unknown. Consequently, tumor-to-tissue ratios may be artificially increased as specific accumulation of [^89^Zr]Zr-DFO-HM30 and [^89^Zr]Zr-DFO-HM34 in nontarget organs may not be accurately captured. Additionally, xCT is highly expressed on immune cells, such as macrophages (32). To visualize interactions with these immune cells, more complex mouse models, such as mice with humanized immune systems (33,34), should be used in future studies.

Theranostics are set to redefine the cancer treatment paradigm. High affinity, specificity, and versatile conjugation methods have made antibodies a popular choice as theranostic agents. In addition to radionuclide therapy approaches, combination strategies with a PET-compatible radioimmunoconjugate for diagnosis and an ADC for therapy hold great promise. We previously demonstrated the capability of the xCT-targeting ADC, HM30-tesirine, to halt tumor growth in xCT-high H460 tumors, whereas growth was unaffected in xCT-low H1299 xenografts (15). Our data establishes [^89^Zr]Zr-DFO-HM30 as a companion diagnostic for xCT-directed therapeutics, such as HM30-tesirine, providing insight into the tumor penetration of these antibody-based agents. [^89^Zr]Zr-DFO-HM30 immuno-PET provides a foundation to develop more effective ADCs and a framework for exploring targeted radionuclide therapy using xCT-directed antibodies. Clinically, these strategies are particularly compelling in NRF2/KEAP1-altered NSCLC, where xCT upregulation drives therapy resistance and underscores the need for targeted interventions. Together, these findings position xCT immuno-PET as both an effective imaging tool for drug development and a gateway to theranostic applications in redox-rewired cancers.

## CONCLUSION

We developed an antibody-based radioligand targeting human xCT, demonstrating specific binding in preclinical models of NSCLC and excellent tumor visualization by PET. This imaging agent provides a direct window into the in vivo biodistribution and tumor-binding abilities of xCT-directed ADC therapies that could improve outcomes in NSCLC.

## DISCLOSURE

This study was funded through a Wellcome Trust Senior Research Fellowship (220221/Z/20/Z) and by U.K. Research and Innovation under the U.K. government’s Horizon Europe funding guarantee (10091247), arising from the European Innovation Council Pathfinder grant (101129886, SMARTdrugs). Abigail Barber is supported by the U.K. Medical Research Council (MRC) Doctoral Training Partnership Studentship (MR/W006820/1). The preclinical PET/CT scanner was also funded by the MRC (MR/X011992/1). No other potential conflict of interest relevant to this article was reported.

## Notes

KEY POINTS **QUESTION:** Can antibody-based PET imaging agents visualize xCT expression and image the in vivo biodistribution of their therapeutic counterparts in preclinical models of NSCLC? **PERTINENT FINDINGS:** [^89^Zr]Zr-DFO-HM30 is an effective imaging tool to visualize the tumor penetration, target binding, and pharmacokinetics of antibody-based xCT-directed therapeutics in NSCLC. **IMPLICATIONS FOR PATIENT CARE:** xCT immuno-PET enables evaluation of next-generation chemotherapeutic and radiotherapeutic strategies incorporating antibodies to target xCT upregulation in NRF2/KEAP1-altered NSCLC.
